# Treated Dentin Matrix in Tissue Regeneration: Recent Advances

**DOI:** 10.3390/pharmaceutics15010091

**Published:** 2022-12-27

**Authors:** Fei Bi, Zhijun Zhang, Weihua Guo

**Affiliations:** 1 State Key Laboratory of Oral Diseases, West China Hospital of Stomatology, Sichuan University, Chengdu 610017, China; 2 Department of Pediatric Dentistry, West China School of Stomatology, Sichuan University, Chengdu 610017, China

**Keywords:** treated dentin matrix, bio-material, bio-induction activity, tissue regeneration

## Abstract

Tissue engineering is a new therapeutic strategy used to repair serious damage caused by trauma, a tumor or other major diseases, either for vital organs or tissues sited in the oral cavity. Scaffold materials are an indispensable part of this. As an extracellular-matrix-based bio-material, treated dentin matrixes have become promising tissue engineering scaffolds due to their unique natural structure, astonishing biological induction activity and benign bio-compatibility. Furthermore, it is important to note that besides its high bio-activity, a treated dentin matrix can also serve as a carrier and release controller for drug molecules and bio-active agents to contribute to tissue regeneration and immunomodulation processes. This paper describes the research advances of treated dentin matrixes in tissue regeneration from the aspects of its vital properties, biologically inductive abilities and application explorations. Furthermore, we present the concerning challenges of signaling mechanisms, source extension, individualized 3D printing and drug delivery system construction during our investigation into the treated dentin matrix. This paper is expected to provide a reference for further research on treated dentin matrixes in tissue regeneration and better promote the development of relevant disease treatment approaches.

## 1. Introduction

Trauma [1], cancer [2], infection [3] and other major diseases [4] are causing serious damage to the human body and even costing human lives. Traditional treatment aimed at tissue damage or defects concentrates on the surgical removal of lesions [5,6,7] and conducting replacements with artificial products [8,9], other parts of the same body [10] or allo- [11]/xeno- [12] organ transplantation, which in some circumstances are considered to have limited effectiveness or present immunogenicity concerns. Fortunately, a new therapeutic strategy was found for this medical problem, that is, tissue engineering [13]. The basis of tissue engineering is to establish a three-dimensional complex of cells and bio-materials that mimic living tissue and can be used to reconstruct the morphology, structure and function of the damaged organ and hopefully achieve permanent replacement.

Scaffold materials are an indispensable part of tissue engineering, among which decellularized extracellular matrix (dECM) bio-materials [14,15] stand out because of their unique natural structure, biological induction activity and bio-compatibility. The dECM-based bio-materials are derived from an extracellular matrix and prepared using various decellularized methods [16]. It can not only provide a three-dimensional scaffold structure for tissue-regenerating cells but also regulate the behavior of these cells [17], including cell morphology, cell adhesion, cell proliferation, cell migration, cell differentiation and apoptosis. In addition, it can induce the proliferation and differentiation of stem cells in the host body and regulate cell signaling pathways and gene expression [18] through mechanical perception or receptor-mediated regulation. There are two types of application strategies used for a dECM. The first one is to directly apply a dECM [19,20] with or without drug molecules, bio-active agents and stem cells to fabricate bio-materials. The purpose is either to promote the recellularization progress [21] of pivotal stem cells in the host body and wait for them to fulfill their mission of differentiating and repairing or to re-perfuse the key stem cells into the dECM, then transplant those as a whole in vivo [22], expecting them to already function from the very beginning. The other one is to see the dECM as the bio-environment for organoids construction [23] or the disease model [24] base for research and sifting drugs.

A treated dentin matrix (TDM) [25] is a special kind of decellularized extracellular matrix (dECM) that originates from a dentin matrix (DM). A TDM preserves the basic structure of natural dentinal tubules, allowing stem cells to adhere and providing the necessary space for nutrient and metabolic waste exchange. It also exposes various and abundant bio-active proteins [26], such as growth factors, to better induce stem cell proliferation, adhesion and differentiation to promote dental pulp, dentin, cementum, periodontal ligament and alveolar bone regeneration [27,28]. Furthermore, a TDM is able to serve as a carrier and release controller for some drug molecules [29,30,31] and bio-active agents [32,33,34] by taking advantage of its unique structure of exposed dentinal tubules. The above characteristics of a TDM have enabled it to become a promising dECM-based bio-material that is capable and worthy of applying to tissue regeneration and related immunomodulation. Although dentin-derived bio-materials have been constructed and explored for potential applications in clinical practice, there lacks a retrospective summary of the overall situation and a prospective discussion regarding future research. Therefore, this review briefly introduces the sources, the fabricating and preserving methods, and the vital properties of a TDM. More importantly, we summarized the applications in the tissue regeneration process and challenges facing future research regarding this promising bio-material.

## 2. The Origin of TDM—Dentin Matrix

Dentin composes the major bulk of tooth hard tissue and serves the functions of protecting dental pulp and supporting enamel. It contains 70% mineral phase; 18% collagen; 2% non-collagenous proteins (NCPs), such as dentin matrix protein-1 (DMP-1), dentin sialoprotein (DSP) and dentinphosphoproteins (DPP); and 10% water [35,36]. The dentin matrix possesses abundant growth factors [33], such as basic fibroblast growth factor, insulin-like growth factor, transforming growth factor β and bone morphogenetic proteins [35], and resembles the bone matrix [26]. In carious circumstances of demineralization, bacteria-produced acid would resolve hydroxylapatite of the dentin matrix, resulting in a loss of highly mineralized particles and degradation of the collagen matrix. However, inspired by the highly pathological process, precise control of the demineralization of dentin matrix acquired from extracted teeth would enable us to achieve the expected objectives of opening the dentinal tubules and releasing the bio-active agents while preserving a certain level of structural integrity and rigidity.

## 3. The Vital Properties of a TDM

### 3.1. Natural Porous Structure Is Preserved after a Fabrication Process and Shows Open Dentinal Tubules

**Figure 1:**
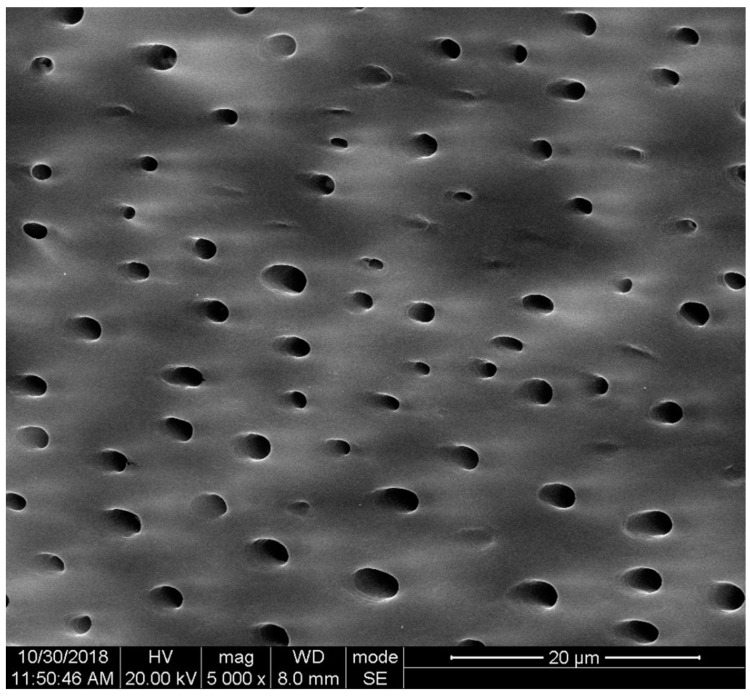
Treated dentin matrix image taken using SEM, where exposed dentinal tubules are shown.

An important property of a TDM is the porous structure, which is present after a serial preparation procedure. This unique natural structure showing exposed dentinal tubules (Figure 1) displays an average diameter of around 2–5 μm [31], which allows nanoscale drug molecules and exogenous bio-active agents to be immersed in and released into the surroundings later. Moreover, this structure supports the nutrient and metabolic waste exchange in the tissue regeneration micro-environment. A root-shaped TDM usually applies to bio-root construction with a 10 mm length and 1 mm thickness, whereas particle-shaped TDM for hydrogel or paste fabrication has a diameter ranging from less than 40 μm to 2 mm [35]. Furthermore, with the prolonging of demineralization time, a treated dentin matrix showed nanoscale fissures among opened dentinal tubules [31], which is also favorable for carrying drug molecules that are to be released later.

### 3.2. A Surface with Proper Hydrophilicity Is Favorable for Cell Adhesion

The proper hydrophilicity of the material’s surface enables either in vitro co-cultured stem cells or in vivo migrating cells to adhere and then proliferate and differentiate on it [30,31].

### 3.3. Odontogenic/Osteogenic-Related Proteins Are Preserved after a Serial Preparation and Are Similar to Those of a Native Dentin Matrix

A dentin matrix contains abundant collagen proteins [37]; non-collagen proteins, such as osteopontin, osteocalcin, dentin matrix protein, dentin phosphoprotein and dentin sialoprotein [38]; and growth factors, such as basic fibroblast growth factor, insulin-like growth factor and transforming growth factor β [35]. It also contains bone morphogenetic proteins (BMPs) [39]. The results of a shotgun proteomic strategy applied to profile human TDM proteome showed that collagens, proteoglycans, small integrin-binding ligand N-linked glycoproteins and growth factors were identified [40], which supplements the evidence showing that a TDM contains a similar protein composition to a native dentin matrix.

### 3.4. Treated Dentin Matrix and Its Derivatives Promote Cell Proliferation on Most Occasions (Table 1)

In most cases, a TDM and its derivatives display a positive effect on the viability and proliferation of co-cultured stem cells [41,42,43,44,45]. Contrarily yet interestingly, sometimes the derivants can display a suppressive or slightly inhibitive effect on cell proliferation [46,47]. Dental pulp stem cells (DPSCs) [41,42,43,44,45,46], odontoblast-like cell lines [42] and periodontal ligament stem cells (PDLSCs) [47] were employed to explore the effect of a treated dentin matrix on cell proliferation by means of an MTT assay, CCK8 assay or Ki-67 detection.

**Table 1:** Treated dentin matrix and its derivatives’ effects on cell proliferation.

Authors Ref.	Type of Cell	Main Conclusion
Brunello et al. [41]	DPSC	Both human dentin particulates and deproteinized bovine bone matrix supported cell proliferation equally well.
Salehi et al. [42]	OD-21 MDPC-23	Dose-dependent promotion of cell proliferation with a higher concentration of the dentin matrix components was verified.
Horsophonphong et al. [43]	OD-21	Both human dentin matrix molecules and bovine dentin matrix molecules enhanced cell proliferation.
Chen et al. [44]	DPSC	Treated dentin matrix paste significantly promoted cell proliferation.
Kulakowski et al. [45]	DPSC	Cells cultured with proanthocyanidin-treated dentin exhibited increased proliferation.
Wen et al. [46]	DPSC	Treated dentin matrix extracts combined with dental-pulp-cell-derived small extracellular vesicles suppressed cell proliferation.
Xiong et al. [47]	PDLSC	Liquid extracts of fresh/cryopreserved/freeze-dried demineralized dentin matrix slightly inhibited cell proliferation.

DPSC: dental pulp stem cell, PDLSC: periodontal ligament stem cell, OD-21: undifferentiated mouse dental pulp cells, MDPC-23: odontoblast-like cell line.

### 3.5. A Treated Dentin Matrix Possesses Osteogenic and Odontogenic Induction Activity (Table 2)

A TDM possesses the ability to induce a couple of mesenchymal stem cells to differentiate toward odontoblasts and osteoblasts. Induced mesenchymal cells can express more odontogenic- and osteogenic-related genes and proteins. Dental pulp stem cells (DPSCs) [33,44,45,48,49,50,51,52], dental follicle cells (DFCs) [27,51,52,53,54,55], cranial neural crest cells (CNCCs) [51], umbilical cord mesenchymal stem cells (UCMSCs) [56] and bone marrow stromal cells (BMSCs) [57] were employed to explore the effect of a treated dentin matrix on cell differentiation by means of polymerase chain reaction, Western blot assay or immunostaining.

**Table 2:** Treated dentin matrix and its derivatives’ effects on cell differentiation.

Authors Ref.	Type of Cell	Main Conclusion
Bakhtiar et al. [48]	DPSC	DMP-1 and DSPP expressions of stem cells increased after treated dentin matrix induction.
Chang et al. [49]	DPSC	Stem cells were positively stained for DSP and DMP1 in the autoclaved human-treated dentin matrix group.
Meng et al. [50]	DPSC	The mRNA expressions of OCN, DSPP, VEGF-1 and Nestin in stem cells were obviously upregulated by a human-treated dentin matrix leaching solution.
Chen et al. [44]	DPSC	Treated dentin matrix paste significantly enhanced the expressions of ALP, BSP and DSP.
Melling et al. [33]	DPSC	Demineralized dentin matrix liposomes promoted the upregulation of OCN and RUNX2 in stem cells.
Kulakowski et al. [45]	DPSC	Proanthocyanidin-treated dentin increased the expressions of RUNX2, BMP2, OCN and DSPP.
Jiao et al. [53]	DFC	A cryopreserved dentin matrix extract liquid induced stem cells to highly express BSP, COL-1 and ALP.
Yang et al. [27]	DFC	Treated dentin matrix induced stem cells to highly express DMP-1 and BSP.
Li et al. [54]	DFC	Porcine-treated dentin matrix can facilitate the odontoblast differentiation of stem cells.
Chen et al. [51]	DFC, DPSC and CNCC	With the induction of a treated dentin matrix, DFCs displayed similar expression patterns of neurofilament, tubulin and nestin to DPCs. Meanwhile, DFCs showed more similar protein profiles of COL1, TGF-β1, OPN and DMP-1 to CNCCs than DPCs.
Zhang et al. [56]	UCMSC	Liquid extract of a human-treated dentin matrix induced stem cells to express DSPP, DMP-1 and DSP.
Yang et al. [57]	BMSC	Human-treated dentin matrix particles promoted the osteogenic differentiation of stem cells.
Yang et al. [55]	DFC and HERSC	A treated dentin matrix’s presence and HERSCs’ induction enhanced the osteogenic differentiation of DFCs.
Guo et al. [52]	DFC and DPC	A treated dentin matrix induced both DFCs and DPCs to display odontogenic differentiation potential.

DPSC: dental pulp stem cell, DFC: dental follicle cell, CNCC: cranial neural crest cell, UCMSC: umbilical cord mesenchymal stem cell, BMSC: bone marrow stromal cell, HERSC: Hertwig’s epithelial root sheath cell.

## 4. Different Methods of Fabricating and Preserving TDM

The fabricating strategy for a TDM usually consists of the following basic steps: teeth extraction, cementum and dental pulp removal, section into matrixes or pulverization into particles, demineralization, special treatment (not always necessary), sterilization and preservation. Grawish et al. offered quite a detailed elaboration on the fabrication process and preservation choices of TDM [35]. Therefore, we intended to focus on some special and novel treatments to facilitate TDM construction or to inspire the improvements of TDM-based bio-material preparation.

### 4.1. Harvesting the Tooth Root from Different Species (Figure 2)

Extracted human teeth are the most common source for harvesting TDM raw material [43,46,49,50,56,57,58,59,60,61,62,63,64,65]. Experiments conducted with animal TDMs, such as bovine [43,48], canine [28], goat [49], monkey [28] and porcine TDMs [28,31,32,46,66,67,68], show that these are also feasible. It is worth noting that the porcine TDM possesses similar mineral phases, bioactive molecules and odontogenic induction ability to a human TDM [66].

**Figure 2:**
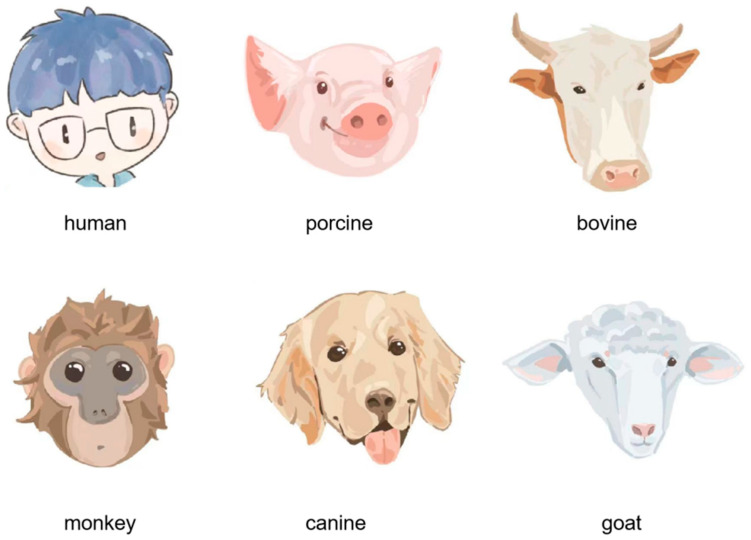
Animal origins of treated dentin matrixes.

### 4.2. Special Treatment Procedures (Table 3)

**Figure 3:**
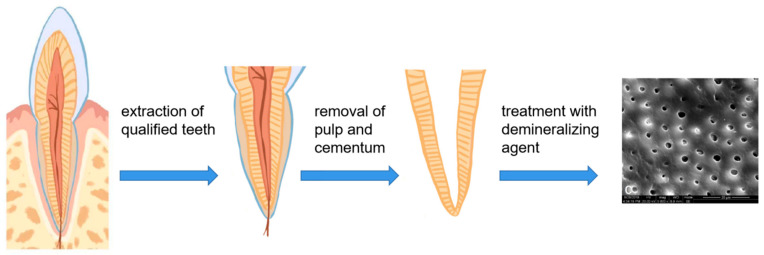
Basic procedure to obtain a treated dentin matrix.

Other than the basic procedures (Figure 3), novel treatments of TDM in various experiments were conducted to explore the possibilities for realizing the goals of decreasing inflammatory responses [48] and promoting dentin–pulp regeneration [58,69]. In addition, some treatment methods for demineralized dentin matrixes are provided as inspiration and references for enhancing the mechanical [59,60] and biological [61] properties of TDMs for use in clinical practices.

**Table 3:** Special treatments of treated dentin matrixes during the preparation procedure.

Authors Ref.	Special Treatment	Main Conclusion
Bakhtiar et al. [48]	Atelopeptidization with pepsin	Atelopeptidization of demineralized dentin could facilitate preserving collagen structure and reducing the immune reaction.
Li et al. [59]	Ethanol/DMA	Treatment using DMA/ethanol solution might be capable of enhancing the mechanical properties of a demineralized dentin matrix.
Omar et al. [60]	Plant-based polyphenols	PB-Ps reduced the degradation of dentin extracellular matrixes and improved the apparent elastic modulus.
Okamoto et al. [61]	MMPs	Dentin matrix components partially digested by matrix metalloproteinases, especially MMP-20, stimulated tertiary dentin formation in vivo and indicated its potential for wound healing of the dentin–pulp complex.
Wang et al. [69]	Freezing	A freeze-dried dentin matrix has similar mechanical and biological properties to those of dentin.
Li et al. [58]	Different grinding speeds	An LTDM induced a twice greater expression of DSPP and DMP-1 in stem cells than an HTDM, while an HTDM induced a twice greater expression of BSP in stem cells than an LTDM. Neo-dentin formed on the inner surface of an LTDM and neo-cementum formed on the outer surface of an HTDM.

DMA: N-(3,4-dihydroxyphenethyl)methacrylamide, PB-Ps: plant-based polyphenols, MMPs: matrix metalloproteinases, LTDM: low-speed hTDM with a maximum speed of 500 rpm, HTDM: high-speed hTDM with a speed of 380,000 rpm.

## 5. TDM Applications

To date, treated dentin matrixes and their derivatives are being employed in the field of tissue regeneration studies, including dental pulp/dentin/dentin–pulp complex regeneration, periodontal tissue regeneration, bone regeneration, bio-root construction and drug delivery system for immunomodulation and regeneration processes. 

### 5.1. Dental Pulp/Dentin/Dentin–Pulp Complex Regeneration (Table 4)

Dental pulp is a connective tissue that contains nerves and vessels inside the chamber formed by dentin and enamel. The objective of dental pulp regeneration refers to vascularized soft tissue with dispersed nerves, orderly odontoblasts contributing peripherally and dentin-like mineralized tissues. Histological detection methods, such as hematoxylin and eosin (H&E) staining, Masson’s trichrome staining and immunostaining are commonly applied to verify the genuine repair outcome. Imaging tests, such as X-rays, cone-beam computed tomography (CBCT) and micro-CT, are employed to examine hard tissue formation. Clinical examinations via the electric pulp test and color Doppler flow imaging (CDFI) are used to evaluate the vitality of pulp tissue. Dental pulp stem cells (DPSCs) [33,49,69,70,71,72]; dental follicle cells (DFCs) [53,65]; and related products [73,74], such as hydrogel [75,76,77], paste [44] and a mixed complex of TDM particles with stem-cell-derived small extracellular vesicles [46], were employed to explore the dental pulp/dentin/dentin–pulp regeneration effect of a TDM or its derivatives.

**Table 4:** Treated dentin matrix and its derivatives for dentin/pulp/dentin–pulp regeneration.

Authors Ref.	Major Composition of the Bio-Material	Main Conclusion
Chang et al. [49]	Allogenous autoclaved TDM + DPSCs	An allogenous autoclaved treated dentin matrix induced stem cells to develop new dentin pulp-like tissues, dental pulp and cementum periodontal complexes. DSP, βⅢ-tubulin, DMP-1, COL-1 and CAP were positive in toothlike tissue.
Liu et al. [70]	TDM + DPSCs	New dentin was found in a rat mandible cultured with a treated dentin matrix and was significantly thicker.
Tran et al. [71]	TD + DPSCs	Treated dentin induced stem cells to regenerate dentin-like tissues that expressed DSPP and DMP-1.
Melling et al. [33]	DDM + DPSCs	Demineralized dentin matrix liposomes increased stem cells’ mineralization.
Wang et al. [69]	FDDM + DPSCs	A freeze-derived dentin matrix supported dentin-pulp-like tissue regeneration, which was positively stained with DSP and ALP.
Liu et al. [72]	DDM + DPSCs	A demineralized dentin matrix could induce DPSC to form mineralized tissue, which was stained positive for DSPP.
Jiao et al. [53]	CDM + DFCs	A cryopreserved dentin matrix could induce stem cells to regenerate new dentin-pulp-like tissues, including dentinal tubules, predentin, collagen fibers, nerves and blood vessels, which were positive for DSPP, DMP-1, tubulin and COL-1.
Li et al. [65]	TDM + DFCs	A human-treated dentin matrix induced complete dentin tissue regeneration that expressed DSP and DMP-1.
Holiel et al. [75,76]	TDMH	A treated dentin matrix hydrogel was developed for direct pulp capping. It could contribute to achieving dentin regeneration and conservation of pulp vitality. CBCT showed TDMH-induced superior dentin bridge formation of higher radiodensity and thickness than Biodentine and MTA. Histological analysis showed TDMH induced thicker dentin with layers of well-arranged odontoblasts than Biodentine and MTA.
Chen et al. [44]	TDMP	A treated dentin matrix paste was developed for pulp capping. TDMP induced the formation of a continuous reparative dentin bridge that was thicker and denser than calcium hydroxide. TDMP achieved both dentin regeneration and vital pulp conservation.
Wen et al. [46]	sEV-TDM	sEV-TDM was developed by combining treated dentin matrix proteins and dental-pulp-cell-derived small extracellular vesicles. It was testified that sEV-TDM promoted the formation of continuous reparative dentin. Odontoblast-like high columnar cells were observed on the pulp side of the dentin bridge.
Cunha et al. [77]	DMM	A microparticulate hydrogel supplemented with dentin matrix molecules was developed for dental pulp capping. A microgel + DMM induced more dentin bridge formation and less pulp necrosis than MTA.
Fu et al. [73]	TDM + DPEM	Treated dentin matrix combined with a laminin-modified dental pulp extracellular matrix promoted odontogenic differentiation of cells and dental pulp regeneration as shown by the expression of DMP-1 and DSPP and a continuous odontoblastic layer-like structure.
Na et al. [74]	TDMF + CSDP + CS	Human-treated dentine matrix fragments combined with a cell sheet and stem-cell sheet-derived pellet induced highly vascularized dental-pulp-like tissue with odontoblast-like cells expressing DSPP, ALP and BSP.

### 5.2. Periodontal Tissue Regeneration (Table 5)

Periodontal tissues include cementum, periodontal ligament, alveolar bone and gingiva. Together, they serve the functions of supporting teeth and rendering the steadiness of teeth. To accomplish the goal of periodontal tissue regeneration, a sandwich structure of cementum-like tissue, bone tissue formation and orderly arrangement of fibers with two ends respectively embedded in cementum-like tissue and buccal bone tissue, resembling Sharpey’s fibers, is required [78]. HE staining, Masson staining and immunostaining are commonly used to verify the fiber and mineralized tissue formation. Imaging tests, such as cone-beam computed tomography (CBCT) and micro-CT are mainly conducted to display the alveolar bone tissue formation outcome. Clinical examinations via the tooth mobility test and probing test are applied to evaluate the healthiness of periodontal tissue. Dental pulp stem cells (DPSCs) [49], dental follicle cells (DFCs) [57,79], stem cells from human exfoliated deciduous teeth (SHEDs) [79] and related products [63,80] were employed to explore the periodontal tissue regeneration effect of TDM or its derivatives.

**Table 5:** Treated dentin matrix and its derivatives for periodontal tissue regeneration.

Authors Ref.	Major Composition of the Bio-Material	Main Conclusion
Chang et al. [49]	Allogenous autoclaved-TDM + DPSCs	An allogenous autoclaved treated dentin matrix induced stem cells to develop cementum periodontal complexes, where COL-1- and CAP-positive stains were produced.
Yang et al. [57]	TDMPs + DFC cell sheets	Human treated dentin matrix particles combined with stem cell sheets induced new bone formation and periodontal-like tissues in animal experiments.
Li et al. [63]	DDM granules	Demineralized dentin matrix granules prepared at the chairside after extractions showed no significant difference in implant stability quotient values and marginal bone resorption when being applied to guided bone regeneration for immediate implantation in periodontal postextraction sites compared with Bio-Oss.
Yang et al. [79]	TDM + DFC TDM + SHED	Treated dentin matrix combined with either DFC or SHED successfully achieved periodontal tissue regeneration, showing periodontal ligament fibers, blood vessels and newly created alveolar bone.
Ji et al. [80]	TDM + PRF	A treated dentin matrix combined with autologous platelet-rich fibrin induced cementum and periodontal ligament (PDL)-like tissue regeneration.

### 5.3. Bio-Root Construction (Table 6)

**Figure 4:**
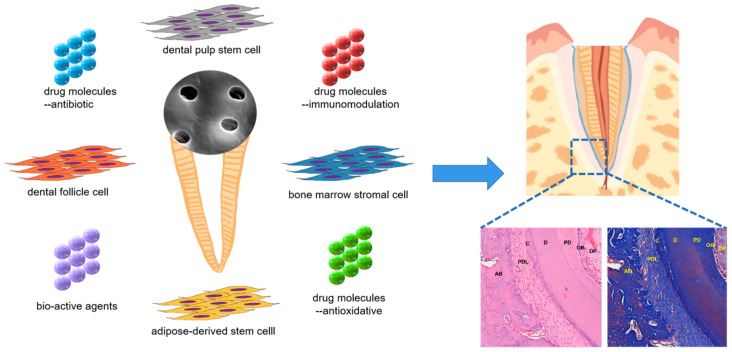
Treated-dentin-matrix-based bio-root construction. AB: alveolar bone, PDL: periodontal ligament, C: cementum, D: dentin, PD: pre-dentin, OB: odontoblast, DP: dental pulp.

TDM-based bio-root construction has not only achieved the histological goal of tissue regeneration regarding pulp, dentin, cementum, periodontal ligament and alveolar bone but has also realized the superior objective of bio-functioning [28], specifically load-bearing masticatory movement [54,81]. Dental pulp stem cells (DPSCs) [50], dental follicle cells (DFCs) [27,28,29,30,31,32,54,66,82], adipose-derived stromal/stem cells (ASCs) [67] and related products [68] were employed to explore the potential of constructing bio-root (Figure 4) out of TDM or its derivatives. The porous structure of a treated dentin matrix has enabled it to become a promising bio-material as a drug carrier and delivery system. The molecules with or without capsule modifications can be detached to the surface or into the tubule chamber of TDM, which are later released from the substance. During research work on bio-root construction, immunological rejection caused by xenograft [30,31,66,83], oxidative stress injury and infection is an inevitably encountered challenge; it could be worth making attempts to load drug molecules of immune response regulators [30,31], antioxidants [29,32], antibiotics [84] and bio-active agents [28,34] for the purpose of achieving better bio-root regeneration. 

**Table 6:** Treated dentin matrix and its derivatives for bio-root construction.

Authors Ref.	Major Composition of the Bio-Material	Main Conclusion
Meng et al. [50]	TDM + Matrigel + DPSC sheet	A treated dentin matrix/Matrigel/DPSC sheet complex was fabricated for promoting periodontium, dentin and pulp-like tissue regeneration. Periodontium-like dense connective tissue, predentin, odontoblast-like cells, blood vessel-like structures and even nerve-like fibers were observed.
Li et al. [66]	pTDM + DFC	A porcine treated dentin matrix induced odontogenesis as observed by the production of pre-dentin, cementum, collagen fibrils, odontoblast-like cells and fibroblasts, even though the xenogeneic implants inevitably initiated Th1 inflammation.
Zhang et al. [29]	TDM + DFC + NAC	A treated dentin matrix loaded with antioxidant NAC decreased HO-induced cellular damage, maintained DFCs’ odontogenic differentiation potential and repressed replacement resorption or ankylosis, thus facilitating bio-root regeneration.
Sun et al. [32]	TDM + DFC + tBHQ	The scaffold of a tBHQ-treated xenogenic treated dentin matrix with DFCs implanted in vivo showed reduced osteolysis and osteoclastic resorption.
Yang et al. [27]	TDM + DFC	A treated dentin matrix induced DFCs to develop new dentin–pulp-like tissues and cementum–periodontal complexes.
Guo et al. [82]	TDM + DFC	A treated dentin matrix induced DFCs to form root-like tissues that were stained positive for markers of dental pulp and periodontal tissues.
Li et al. [54]	TDM + DFC	A porcine treated dentin matrix combined with DFCs was transplanted into the jaws of rhesus monkeys. Periodontal ligament-like fibers accompanied by macrophage polarization, fibroblasts and blood vessels were observed. Meanwhile, the constructed bio-root possessed biomechanical properties that could endure masticatory forces.
Guo et al. [52]	TDM + DFC/DPC	A treated dentin matrix induced both DFCs and DPCs to form pulp–dentin/cementum–periodentium-like tissues.
Chen et al. [28]	TDM + DFC + BMP4 + TGF-β1	A treated dentin matrix combined with TGF-β1, BMP4 and DFCs embodied a spatial interface gradient for functional enthesis formation to promote functional bio-root regeneration. Effectively functional bio-roots made of the composites were successfully constructed, with the presentation of outstanding biomechanical properties and healthy gingiva.
Luo et al. [81]	CAD- and FEA-based shape-optimized TDM	Computer-aided design and finite element analysis were used to create shape-optimized treated dentin matrix combined with stem cells that successfully achieved root regeneration and a stable performance of masticatory function.
Yuan et al. [67]	TDM + ASC	A porcine treated dentin matrix induced ASC to differentiate toward odontogenesis and promoted dentin-like tissue, pulp-like tissue and periodontal-fiber-like tissue regeneration.
Chen et al. [68]	TDM + APES + DPEM	A treated dentin matrix combined with an aligned PLGA/gelatin electrospun sheet and dental pulp extracellular matrix promoted pulp–dentin complex-like tissues and periodontium-complex-like tissue regeneration, presenting columnar odontoblast-like cells, newly formed predentin, blood vessels, cellular cementum and periodontal ligament (PDL)-like tissues.
Han et al. [31]	NDM + RSG	A native decellularized matrix made of treated dentin matrix loaded with RSG decreased the expression of IL-1 and TNF-α, increased the expression of IL-10 and TGF-β, induced M2 macrophages to antagonize M1 macrophages using PPARγ, created favorable immunomodulation and promoted ligament-to-bone regeneration.
Lan et al. [30]	ECM + RSG	Extracellular matrix made of treated dentin matrix loaded with RSG activated PPAR-γ downregulated the expression of proinflammatory NOS2 + M1 macrophages and ROS to facilitate bio-root regeneration.
Li et al. [83]	TDM + PPARγ-primed CD68CD206 M2 phenotype	A treated dentin matrix combined with PPARγ-primed CD68CD206 M2 phenotype alleviated proinflammatory cytokines (TNF-α, IFN-γ) at the inflammation site; decreased CD3CD8 T lymphocytes in the periphery system; immunosuppressed IL-1β, IL-6, TNF-α and MMPs; enabled xenograft escape immune rejection; and promoted a xenogenic bio-root to survive in the host.

NAC: N-acetylcysteine, tBHQ: tert-butylhydroquinone, TGF-β1: transforming growth factor beta 1, BMP4: bone morphogenetic protein 4, CAD: computer-aided design, FEA: finite element analysis, RSG: Rosiglitazone, PPAR-γ: peroxisome proliferators receptor γ, ROS: reactive oxygen species.

### 5.4. Bone Regeneration (Table 7)

Treated-dentin-matrix-based bio-material’s application in bone tissue regeneration mainly focuses on alveolar ridge preservation and socket preservation after extraction [62,64,85,86,87]. HE staining, Masson staining and immunostaining are commonly used to verify the mineralized tissue formation. Imaging tests, such as cone-beam computed tomography (CBCT) and micro-CT, are mainly conducted to display the alveolar bone tissue formation outcome.

**Table 7:** Treated dentin matrix and its derivatives for bone regeneration.

Authors Ref.	Major Composition	Main Conclusion
Moraes et al. [85]	DHDM	Demineralized human dentine matrix contributed to alveolar ridge preservation, as testified by microtomography and histological evaluation showing new bone formation with the slow reabsorption of DHDM.
Li et al. [86]	ADDM	Autogenous demineralized dentin matrix exhibited osteogenic effectiveness in bone augmentation, just as Bio-Oss^®^ did for oral bone defects.
Murata et al. [62]	pDDM	Partially demineralized dentin/cementum matrix contributed to socket preservation as shown by bone-like radio-opacity in the graft region and newly formed bone connected directly with dentin/cementum area.
Um et al. [87]	aDDM	Autogenous demineralized dentin matrix loaded with recombinant human bone morphogenetic 2 contributed to socket preservation. BMP-2 enhanced bone formation effectiveness.
Reis-Filho et al. [64]	DHDM	A human demineralized dentine matrix has the potential for osteogenic induction and increases bone tissue formation and vessel tissue formation in sockets.

## 6. Future Perspectives

### 6.1. Underlying Mechanism

Despite the fact that there has been a lot of research aimed at application exploration and induction ability confirmation, only very few studies have paid attention to the underlying mechanisms [70]. In this case, it might hinder us from digging more deeply for knowledge of TDMs’ biological induction abilities. Therefore, in-depth studies about mechanisms are needed. 

### 6.2. Expanding Sources

In most cases, we employ the intact teeth without any pathological condition for TDM fabrication and apply it to tissue regeneration. However, we must not ignore the huge potential for extracted teeth subjected to dental diseases, such as caries and periodontitis. It is worth conducting experiments to examine the physical properties and biological abilities after the same fabricating procedure (with and without the action of removing the obvious lesion) to explore and expand the sources that are already regarded as medical waste yet possess great possibilities for recycling.

In order to further expand the source of TDM raw materials, animal teeth are eligible and cost-efficient to be taken into consideration for massive production. This indeed puts forward serious concerns about the similarities between an animal TDM and human TDM and xenogeneic-substance-related immune response regulation. Porcine TDM was already testified to possess similar mineral phases and bio-active molecules to a human TDM [66]. Immunomodulation-related drugs are also being investigated regarding alleviating the immune response of xenogeneic TDM transplantation and facilitating bio-root tissue regeneration and functional stability [30,31]. It is necessary to continue launching experiments to compare the TDM properties between more species. Meanwhile, it is also very important to carry out studies on immunomodulation to create a xenogeneic-friendly micro-environment for a xenogenous bio-root to survive.

### 6.3. Three-Dimensional Construction for Individual Customization in Clinical Application

When it comes to clinical application, the shape and size of damaged tissue vary from patient to patient. Therefore, in order to obtain a precise match between transplantation and the defective tissue region, three-dimensional bio-printing is available. A TDM particle can be pulverized into micron-order particles and mixed into the bio-ink of a 3D bio-printer to harvest the best morphological match and achieve an individualized repair goal [88].

### 6.4. Taking Better Advantage of the Natural Porous Structure for Drug Delivery

Last but not the least, the naturally porous structure of open dentinal tubules are showing great potential for drug delivery, yet is only moderately being taken advantage of. On the one hand, the porous structure of the material and the collagen fibers exposed by decellularization also provide the material basis for drug delivery [31]. On the other hand, there are many active groups on the surface of a treated dentin matrix that also provide favorable conditions for drug loading [54].

In addition, considering that drug molecules delivered by bio-materials are always applied to tissue regeneration with a combination of stem cells, it is also of necessity and importance to notice the interactions between the nanoparticles and the vital cells in the biological environment to achieve a favorable therapeutic effect. Specifically speaking, in the research fields of root canal sealing agents application [89], periodontal tissue regeneration [90] and endodontic disease treatment [91], studies concluded that most of the drug delivery systems provide a micro-environment for tissue remineralization while also displaying a beneficial effect on coupling stem cells, avoiding cytotoxic disadvantages and offering benign biocompatibility, which the treated dentin matrix can also achieve. 

Moreover, proteins in blood and tissue fluid would be adsorbed onto the surface of the material to form a protein layer within a few nanoseconds of contacting the bio-material. This protein layer constitutes a temporary structure that recruits inflammatory-response-related cells and initiates the acute immune inflammatory response [92]. Likewise, a protein-rich layer called the “protein corona” coating on the nanoparticles’ surface would form after interaction with the surrounding biological environment [93]. In order to achieve the goal of balancing the immune responses and constructing a micro-environment that is favorable for promoting tissue regeneration, the characteristics and regulating strategies of these two structures of the protein layer are not to be neglected. 

To date, there are only a few studies that reported a TDM carrying anti-oxidant or immunomodulation drugs [29,30,31] and bio-active agents [32,33,34] via a very rigid loading strategy of just soaking a TDM into the drug solution. Therefore, in the future, more studies of drug molecules or bio-active agents carried by a TDM with or without bio-modification are needed to explore the effects of themselves and TDM delivery efficiency during the regeneration process. Furthermore, the methods of loading molecules and agents into or onto the scaffold should be modified and innovated. 

## 7. Conclusions

A TDM serves as a promising candidate for bio-root construction due to its natural characteristic of being able to induce dental pulp cells to differentiate into odontoblasts and form dentin-like tissues, as well as to induce dental follicle cells to differentiate into cementoblasts, fibroblasts and osteoblasts to form cementum-like tissues, periodontal-ligament-like fibers and bone tissues. In the meantime, a TDM can also be ground and sifted to micro-sized particles and mixed with other materials to become bio-ink for 3D printing, pulp capping agent and osteoinduction supplements. Other than the abovementioned, TDM extracts are also highly bio-active and osteoinductive agents that can be applied to tissue repair [43,61]. What is noteworthy is that the application of TDM will not be restricted to the field of just dental research. Thanks to the unique porous structure and growth factors preserved within, a TDM is able to play a role in bone regeneration, soft–hard tissue interface reconstruction and immunomodulation with its own endogenous bio-active molecules and carried exogenous drugs [29,30,31,32]. Furthermore, the sources of TDM include homogenous human teeth extracted due to orthodontics needs and heterogenous extracted animal teeth from wasted jawbones, which are sufficient and ethically friendly.

Thus, it is necessary to provide a more solid theoretical basis for tissue regeneration by taking advantage of TDM by performing deeper probation into all levels of mechanisms from molecules to tissues, from signaling pathways to function performances and from micro-environment to structural construction. Additionally, it is urgently needed to put forward serious challenges facing clinical translational application to impel researchers to contribute realistically to constructing genuinely useful clinical practice that is beneficial to humankind.

In summary, a treated dentin matrix not only plays an important role in tooth and periodontal repair but also has the potential to promote other tissue regeneration. Despite the fact that considerable work has been done in the field, specific regeneration processes, underlying mechanisms and clinical application possibilities still need to be further explored and illustrated.

## Data Availability

The relevant data is contained within this review.
